# Health and the Megacity: Urban Congestion, Air Pollution, and Birth Outcomes in Brazil

**DOI:** 10.3390/ijerph19031151

**Published:** 2022-01-20

**Authors:** Marcos A. Rangel, Romina Tomé

**Affiliations:** 1 Sanford School of Public Policy, Duke University, Durham, NC 27708, USA; marcos.rangel@duke.edu; 2 American Institutes for Research, Arlington, VA 22202, USA

**Keywords:** air pollution, inversions, birth outcomes, environmental health, semiparametric estimation, Brazil

## Abstract

We studied the health effects of economic development in heavily urbanized areas, where congestion poses a challenge to environmental conditions. We employed detailed data from air pollution and birth records around the metropolitan area of São Paulo, Brazil, between 2002 and 2009. During this period, the megacity experienced sustained growth marked by the increases in employment rates and ownership of durable goods, including automobiles. While better economic conditions are expected to improve infant health, air pollution that accompanies it is expected to do the opposite. To untangle these two effects, we focused on episodes of thermal inversion—meteorological phenomena that exogenously lock pollutants closer to the ground—to estimate the causal effects of *in utero* exposure to air pollution. Auxiliary results confirmed a positive relationship between thermal inversions and several air pollutants, and we ultimately found that exposure to inversion episodes during the last three months of pregnancy led to sizable reductions in birth weight and increases in the incidence of preterm births. Increased pollution exposure induced by inversions also has a significant impact over fetal survival as measured by the size of live-birth cohorts.

## 1. Introduction

Congestion of urbanized centers is a trademark of economic development across the globe. These economic forces pose a challenge with respect to environmental conditions, highlighting a trade-off between economic and physical well-being. While empirical evidence has helped shape environmental regulation in developed nations, there is a scarcity of evidence to inform policy-makers in the developing world [1,2,3]. We aim at filling this gap in the literature.

In this paper, we undertook a causal inference study of urban air pollution’s impact on the health of infants *in utero* in a metropolitan area of Brazil. The identification of this effect is challenging for many well known reasons, such as families’ self-selection into residential locations, and the direct link between economic activity and pollution. To address these concerns, we took advantage of the high-frequency meteorological phenomenon of thermal inversion, which, in urban areas arguably exogenously locks pollutants closer to the ground—increasing exposure among urban dwellers. Relevant for our identification strategy, the formation of thermal inversions does not have any direct effect on health conditional on weather conditions, nor is it easy to predict or anticipate (beyond seasonal patterns).

Our empirical analysis has two parts. We focus first on understanding the effect of thermal inversion on air pollution. Using unbalanced panel data on concentrations of five pollutants and weather conditions between the years 2001 and 2009, we explore how the concentration of pollutants increases with the frequency of inversion episodes. We find that an additional thermal inversion during a seven-day period (mean 2.8; standard deviation [S.D.] 2.0) increases the average concentration of particulate matter under 10 micrometers (PM_10_), carbon monoxide (CO), and sulfur dioxide (SO_2_) by 1.5 to 2.0% of their average weekly concentration. Our data also reveal that these pollution effects from a thermal inversion are equivalent to the effect of an increase of about 30% in vehicular traffic sluggishness within the area. Finally, we show that when considering dynamic effects of inversions, these impacts last up to three weeks and can be more than twice as large as the contemporaneous effects.

Our second set of estimations examine the reduced-form link between inversion episodes and health at birth. By employing detailed data from vital records for the universe of mothers residing within the São Paulo metropolitan area (SPMA), we estimate cumulative effects of air pollution on infants’ health at birth. Our model flexibly accounts for potential effects of weather and seasonality on health. We find that exposure to thermal inversion episodes harms health at birth. Specifically, one additional inversion per week within the last 13 weeks of gestation leads to a decrease in birth weight of 23.3 grams, and an increase of 0.6 percentage point in the incidence of low birth weight (or 7.3% relative to the average). These effects are partially explained by a reduction in gestational length: the incidence of preterm and very preterm births increases significantly with the number of inversion episodes. We also find that the same increase in the frequency of inversions leads to a sizable 18.5% reduction in birth cohort size that affects both male and female fetuses. Because of the latter, we expect that our main effects in terms of birth outcomes represent a lower-bound for the impact of pollution on infant health, since pregnancies of stronger fetuses are likely the ones which end up being captured in observed live births.

Our work builds on [1,4], who studied the contemporaneous effect of pollution on infant mortality in Mexico City and infants’ respiratory problems in Sweden, respectively. We complement these studies by examining the effects of prenatal exposure to inversion episodes on birth outcomes. Because health early in life is a predictor of future outcomes, such as health, earnings, and education [5,6], our results suggest lasting negative consequences of air pollution on new generations and call attention to improved policies oriented to reduce pollution as well as those oriented to provide quality prenatal care within the megacity.

## 2. Background: Air Pollution in the SPMA

### 2.1. Sources of Air Pollution

The SPMA is one of the largest urban conglomerates in the world; its 39 municipalities extend over 8000 square kilometers (km${}^{2}$) and house more than 20 million people. The municipality of São Paulo, with 11 million people, is in the center of this dynamic urban area. Two main sources of pollution contribute to the deterioration in air quality in the SPMA: the large fleet of vehicles and industries with high polluting potential [7]. Studies commissioned by the state’s environmental authority indicate that by 2009 motor vehicles were responsible for 97% of the emissions of carbon monoxide (CO), 96% of nitrogen oxides (NO_x_), 40% of particles matter under 10 micrometers (PM_10_), 97% of hydrocarbons (HC), and 32% of sulfur dioxide (SO_2_) [7]. Then, by reacting with sunlight, HC, NO_x_, and other volatile organic compounds contribute to the formation of ozone (O_3_) [8]. Industrial processes account for most of the rest of these pollutants’ emissions [7]. The SPMA is a large industrial area containing about 2000 pollutant industries (mainly food, chemical, and oil industries) [9]. We studied the period immediately preceding the one from which these pollution sources’ breakdowns are computed.

**Figure A1:**
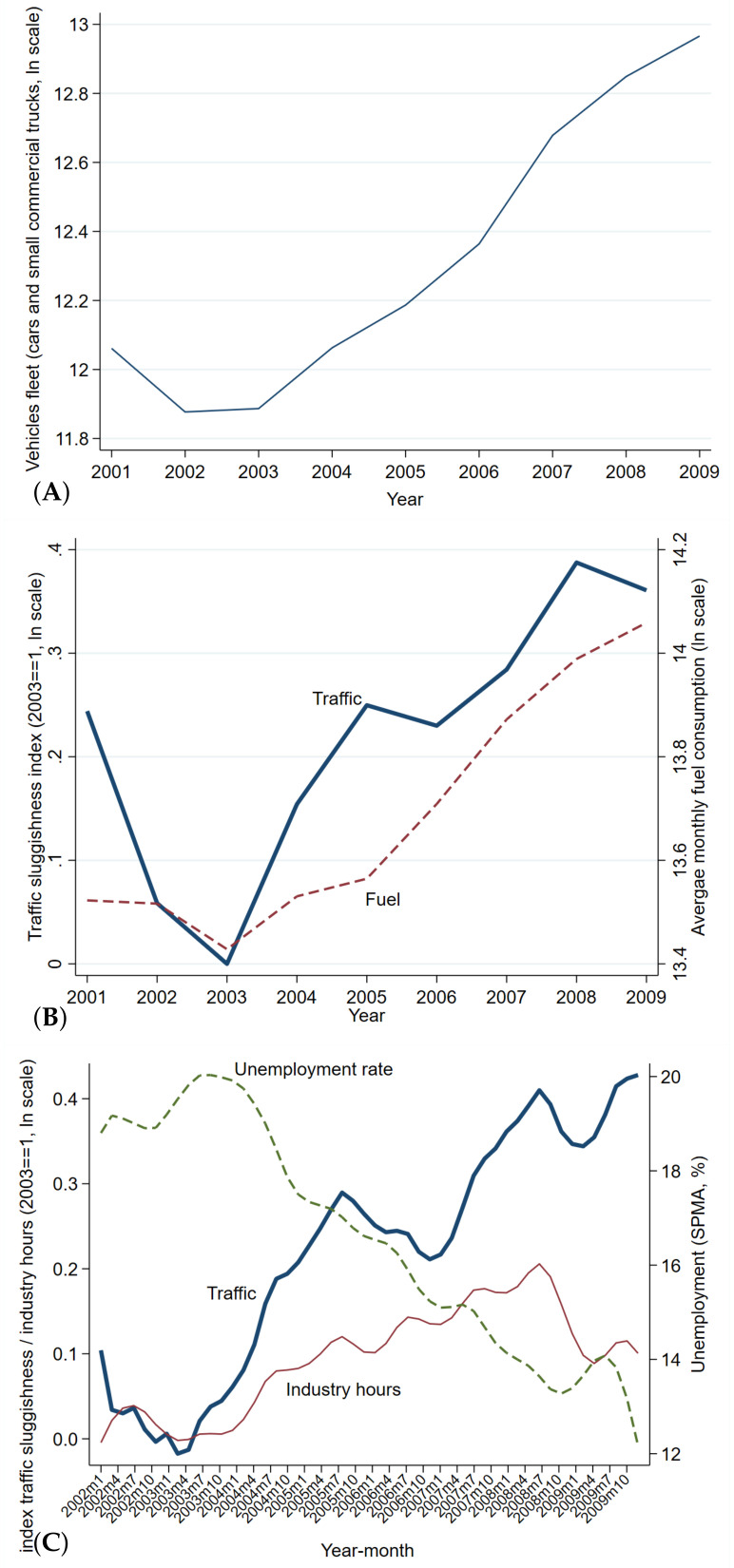
(**A**) Trends in fleet, (**B**) traffic and fuel consumption, and (**C**) economic activity–SPMA.

The trajectories observed between 2001 and 2009 are particularly interesting for our empirical exercise. Fueled by a nationwide economic expansion that increased average income and expanded access to credit, the number of vehicles in the area augmented significantly, from 6 to 9.7 million, or from 331 to 492 vehicles per 1000 inhabitants (see Panel A in Figure A1). Because this expansion was unmatched by infrastructure investments within the SPMA [7,9], such a large number of private vehicles translated into a mobility crisis—the daily average traffic jams during rush hours in the city of São Paulo reached 118 km (112 miles), with cars circulating with an average speed of 19.3 kilometers per hour (km/h). On average, *paulistanos* wasted almost one month per year trapped in traffic [10]. Panel B in Figure A1 displays the time evolution of traffic sluggishness as well as total fuel consumption. Therefore, economic improvements, which likely foster better birth outcomes, were accompanied by environmental risks in the form of urban congestion (i.e., vehicular fuel burning-related emissions) that create well known challenges for fetal development. We reproduce the same relation between economic activity and traffic sluggishness with higher-frequency data from the SPMA in Panel C of Figure A1.

**Figure A2:**
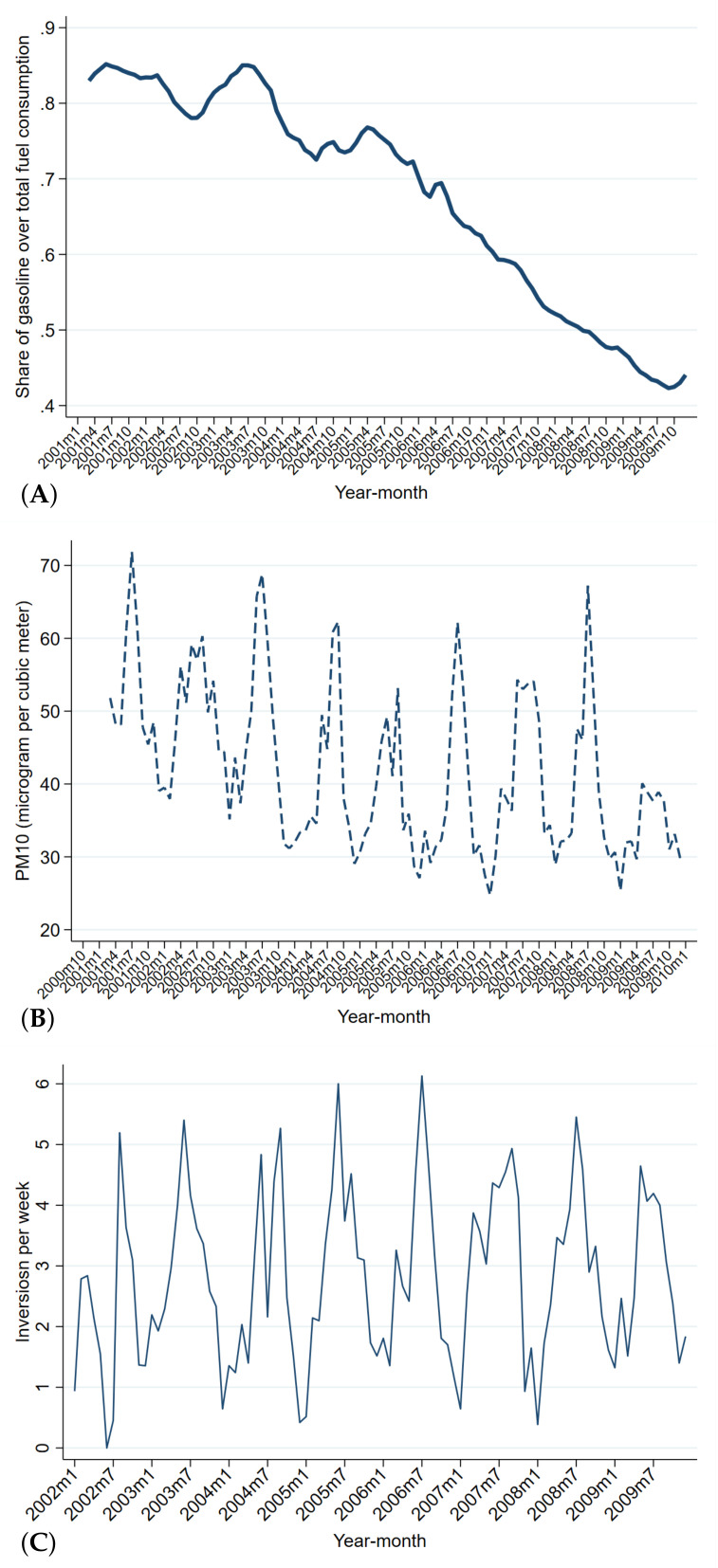
(**A**) Trends in gasoline consumption, (**B**) particulate matter concentrations, and (**C**) weekly thermal inversions–SPMA.

Interestingly, the secular trend in increased traffic and potential increased emissions from the larger fleet was accompanied by a change in the quality of the fleet. While new cars are expected to pollute less than the older ones they substitute, in the SPMA there was also an increase on the adoption of flex-fuel cars that can also run on sugarcane-based ethanol (which is a cleaner option). International prices of oil and sugar together with domestic subsides for the production of ethanol have promoted this pattern. Figure A2 (Panel A) reproduces fuel utilization patterns throughout the state of São Paulo (mostly driven by SPMA consumption patterns) over the period studied. We capture the transition from more than 80% of the fuel consumption being based on gasoline in 2001 to around 40% by 2009. Overall, these trends co-existed with a slight reduction in pollution levels, PM_10_ (Panel B, Figure A2), suggesting that the fleet volume effects were counterbalanced by the fleet composition effects. Panel C in Figure A2 shows that the seasonal pattern of weekly thermal inversion formation that accompanied these changes over time remained relatively stable between 2001 and 2009.

### 2.2. (Lack of) Dispersion of Pollutants: The Role of Thermal Inversions

The concentration of pollutants in the atmosphere depends on the amount of pollutants emitted by the sources (as discussed in Section 2.1) and on the prevailing weather conditions. In the SPMA, meteorological conditions are unfavorable to the dispersion of pollutant: during winters, there are frequent low altitude thermal inversions, and during other seasons, strong solar radiation occurs. The topography of the SPMA contributes to the deterioration of air quality since the area is formed by floodplain surrounded by mountains in the north and northwest, receiving predominant winds from the ocean at southeast [11].

The formation of low-level thermal inversions is a key condition in hinder the dispersion of pollutants. Normally, temperature in the troposphere decreases with height; thermal inversion layers occur when temperature increases with altitude, resulting in a mass of hot air on top of a mass of cold air. During an inversion, the coldest, densest air is at the surface, and its density steadily decreases with increasing height. The stable stratification of air resists uplifting of the particles from surface to atmosphere because thermal inversion layers act like a cap in which vertical air movement remains almost nil and pollutants are trapped close to the ground [4,12].

The formation of inversions is more common during nights when cold ground temperatures cool the air that is closer to the ground and creates warm air over cold air [13]. Roughly speaking, the diurnal cycle of inversions starts after sunset when sunlight intensity is negligible, temperature decreases, and ground (or initial elevated) inversions form [14]. During night, when temperature decreases, these inversions strengthen. They are strongest at sunrise when surface temperature begins to increase from overnight minimum, and they become weaker once the warmer ground warms the air. By midday inversions are completely burned off. Wind speed and rain also contribute to weaken inversions [15]. Because the dynamics of these inversions are driven by the heating of the surface, episodes become more frequent (and last longer) in winter when day lengths are shorter, sun angles lower, and surfaces wet or frozen; summer speeds up the process of breaking inversions [16].

The shape of the landscape also has a role in the formation, lifetime, and intensity of inversions [12]. In valleys, denser and heavier cool air flows down the slopes and settles under warm air leading to stronger effects. Inversions can be produced when a layer of cool air descends through a layer of hot air from vertical air movements or from horizontal movements of air at different temperatures [13].

There are two factors of the formation of inversions that are worth noting for our study. First, the formation of thermal inversions is independent of the current economic activity (including industrial activity and traffic) conditional on weather variables. In the longer run, urbanization influences the heating of the surface and wind, and it may affect both formation and breakup of inversion episodes [12]. Yet, these changes take many years. Ref [17] study urbanization and climate change in the SPMA for the period 1930–2015 and show that the average of maximum temperature increases up to 1.1 Celsius every 20 years.

Second, the meteorological factors associated with thermal inversions (e.g., temperature and relative humidity) may directly impact health and spread of airborne diseases, such as influenza. However, conditional on weather conditions and air pollution, thermal inversions do not represent a health risk by themselves. In our empirical design, it is the increased exposure to pollution that occurs when there is an inversion episode that is likely to be risky for human health.

## 3. Prenatal Exposure to Air Pollution

Pollution may affect infants’ health while in the womb, since this time constitutes a state of high level of cell proliferation, organ development, and the changing capabilities of fetal metabolism [18]. While the exact mechanisms linking exposure to air pollution and fetal development has not been identified yet, studies suggest that direct and indirect effects may be at play [19,20,21,22,23]. Direct effects relate to the translocation of particles across the pulmonary barrier to the blood circulation and then to the placenta, allowing pollutants to reach the fetus; inflammatory and oxidative responses drive adverse effects. Indirect effects may arise from translocated particles causing placental dysfunction or the activation of inflammatory and oxidative responses in maternal lung leading to circulating inflammatory mediators, which affect the placenta, the fetus or the ability of the mother-to-be to carry a fully healthy pregnancy.

Controlled animal studies have shown that pulmonary exposure to pollution, and, in particular, to traffic-related air pollution or diesel exhaust, during pregnancy, leads to abnormal deliveries (including abortion) and affects gestational length, number of offspring, fetus weight, and gender ratio [20,24,25]. In the context of this study, São Paulo’s urban levels of air pollution have been linked to problems in the lung developments of mice [26,27]. Human epidemiology studies document an association between prenatal exposure to pollution from traffic and industries and adverse birth outcomes, such as low birth weight and preterm birth (for a summary of the literature see [28,29,30]), as well as the risk of spontaneous abortion and of stillbirth [31,32,33]. Research focused on São Paulo’s population arises to similar conclusions (see, for example, [34,35,36,37]). After birth, air pollution may cause several health issues, such as respiratory and cardiac troubles. These later life effects may be influenced by the consequences of *in utero* exposure to pollution that make children more vulnerable, yet there is no clear understanding of the functional form or magnitude of these interactions [38].

Epidemiology evidence based on cross-sectional, cohort, or control-case studies fails to account for several sources of confounding effects, including families’ residential sorting and avoidance behaviors. If those who prefer to live in cleaner places (or adopt averting behaviors) are also healthier, wealthier, have greater access to healthcare, or make higher quality investments in their children, study results would be biased upward. These studies also do not account for the fact that urban air pollution is correlated with economic activity, and the latter affects infant health directly. Aiming to overcome these challenges, a growing body of the economic literature has employed quasi-experimental methods to show that pre- and post-natal exposure to urban air pollution has adverse effect on infants’ health at birth and after birth (e.g., [39,40,41,42,43]). These authors provide evidence of the effects on health in developed countries, but their implications for urban air pollution in developing countries remain unclear. The differences in air pollution levels and a population’s health between developed and developing nations restrict the extrapolation of results.

There is a scarcity of studies using applied microeconometric techniques in developing countries, mainly because of data limitations (see, for example, [1,2,3]). One of these few papers instruments air pollution with thermal inversion episodes in Mexico City [1]. Linking the number of thermal inversions per week, weekly pollution levels, and contemporary infant mortality, the authors show that increasing PM_10_ or CO by 1% raises infant mortality by 0.42% or 0.23%, respectively. Our work builds on this study, and, as an extension, we explore the effects of pollution on fetal development contributing to understand how effects after birth differ depending on the level of exposure before birth. Unlike the authors, we use thermal inversions to address the well known concerns related to estimating the effects of air pollution on health taking a more conservative approach: to minimize the difficulties that the lack of data on all pollutants brings, we employ inversion episodes in an intention-to-treat analysis (a strategy also used by [4] to explore exposure after birth and children’s respiratory health problems).

## 4. Data and Summary Statistics

We merged several datasets: air pollution data, weather data, thermal inversion data, and birth records. The latter corresponds to the SPMA’s universe of births between 2002 and 2009; the others correspond to the period from April 2001 to December 2009, allowing us to recover the conditions during *in utero* time for all newborns.

### 4.1. Pollution

**Figure A3:**
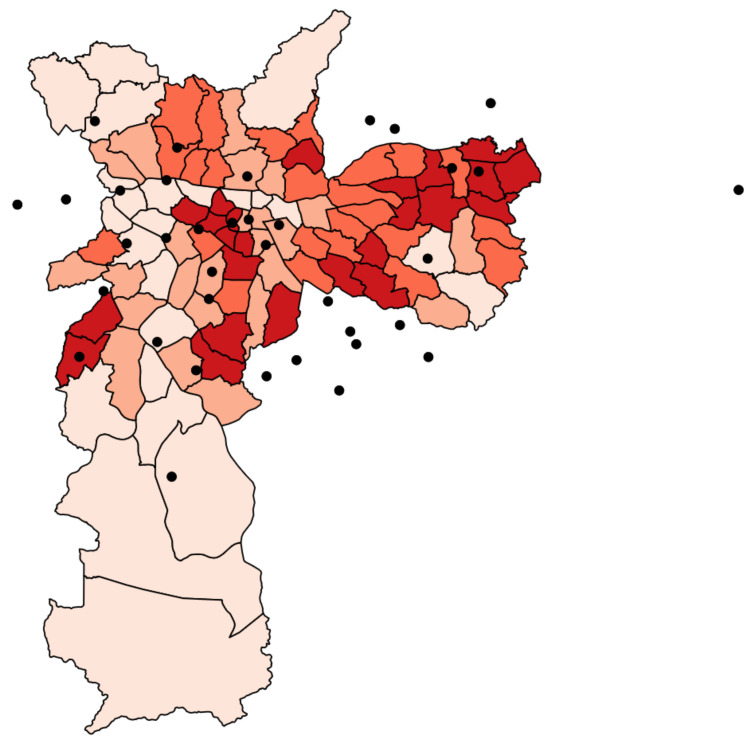
City of São Paulo districts (polygons) and pollution/whether stations (points)–darker shades represent districts with denser populations.

**Figure A4:**
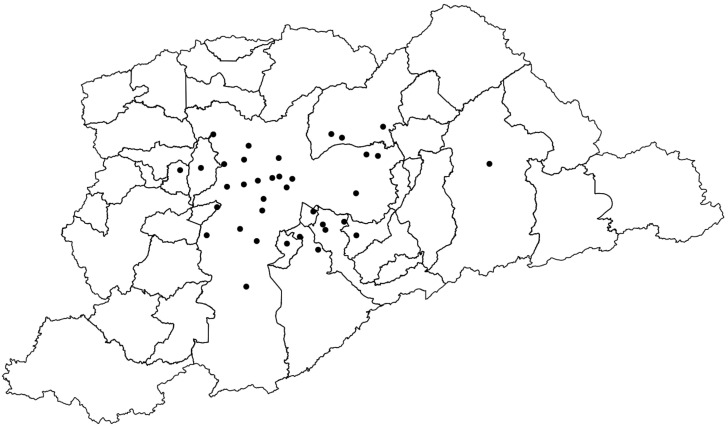
Metropolitan Region of São Paulo municipalities (polygons) and pollution/whether stations (points).

We studued five pollutants: PM_10_, CO, O_3_, NO_x_, and SO_2_. Data came from air monitoring stations operated by the State of São Paulo’s environmental agency *Companhia Ambiental do Estado de São Paulo* (CETESB). For the period, 2001–2009, PM_10_ data were available for 25 stations; CO was collected in 16 stations; O_3_ data were recovered in 15 stations; NO_x_ data were drawn from 12 stations; and SO_2_ information was available for eight stations. None of these were provided in an unbalanced panel data format, being subject to missing data issues. Figure A3 and Figure A4 present the maps of the study area and locations of pollution/weather stations.

Data are organized in hourly observations of pollution concentrations at the station level. For all pollutants (except CO), we calculated daily average concentrations of pollutants for every day, with at least eight hours of raw data. For CO, we used the hourly observations to calculate the maximum daily eight-hour average, which corresponded to the metric used for the US ambient air quality standard [44,45]. Then, for all pollutants, we computed “rolling week” averages. That is, for each date *t*, we computed the average of the daily measure from t−6 to *t*.

The top panel of Table A1 presents descriptive statistics of the concentration of pollutants. Mean levels of PM_10_, CO, and SO_2_ in the SPMA are higher than in California and Sweden, but lower than in Mexico City. The average level of PM_10_ in our data reached 41 μg/m${}^{3}$ whereas it was below 30 μg/m${}^{3}$ in California and Sweden [4,42] and around 67 μg/m${}^{3}$ in Mexico City [1]. The mean levels of O_3_ in the SPMA are similar to those in Mexico City (around 33 μg/m${}^{3}$).

### 4.2. Weather

Weather conditions are key elements in the analysis because they are directly related to thermal inversions, economic activity, and health. Temperature, relative humidity, and wind data are collected by CETESB; rainfall data come from the São Paulo department of water and energy *Departamento de Águas e Energia Elétrica* (DAEE). We converted daily hourly observations of weather variables (except wind) into daily averages if there were at least eight hours of raw data for that date. For wind, we considered the daily prevailing wind as the measure of daily summary. From raw data that were coded as angles in degrees (i.e., 0 indicates wind from due North, and 180 corresponds to wind from due South), we defined octants as the sector of the wind rose and compute frequencies.

Not all CETESB stations that collect air pollution data compute weather data, and some stations that provide weather data do not measure the concentration of pollutants. For those stations collecting only pollution data, we constructed weighted daily averages of weather covariates employing data from stations within a radius of 20 miles. We used the inverse of the distance between stations as weights. Using daily (interpolated) data, we computed rolling week averages by station. Panel B of Table A1 presents mean and S.D. of weather covariates based on observations for all stations.

### 4.3. Thermal Inversions

To trace thermal inversion episodes we used diurnal vertical temperature profile data collected by CETESB and the University of Wyoming. Balloons were launched at 12 UTC (10 am in Brazil), from one central location, to gather temperature, humidity, and wind data, as they ascended through the troposphere. We coded boundary layer inversions every time a temperature at a given altitude was warmer than the temperature at an altitude below it; the opposite non-monotonic temperature gradient allowed us to identify the top of an inversion.

In 7.2% of the dates in our analysis, thermal inversion data were not collected. That is, in 29% of the weeks, there was at least one date without inversion data; dates with missing data represent, on average, less than a date per week (Table A1, Panel C). We took a conservative approach to deal with these missing values when constructing rolling week averages: we assigned a zero to each rolling week with at least one date with missing information, and we controlled semi-parametrically in our specification for the number of missing values in each week.

More than one inversion can be found each day at different altitudes. We focused on inversions that occurred closer to the ground; that is, inversions found below 1.331 m from the sea level. The 1.3 km limit was based on two facts: the station collecting data was located 731 m above sea level, and inversions that occurred closer to the ground generally controlled ground-level pollutant concentrations [46]. We considered inversion episodes that occurred above the station level and, following previous papers in the literature, we used 600 m above the ground to characterize these inversions [4].

### 4.4. Inversions and Weather

**Figure 1:**
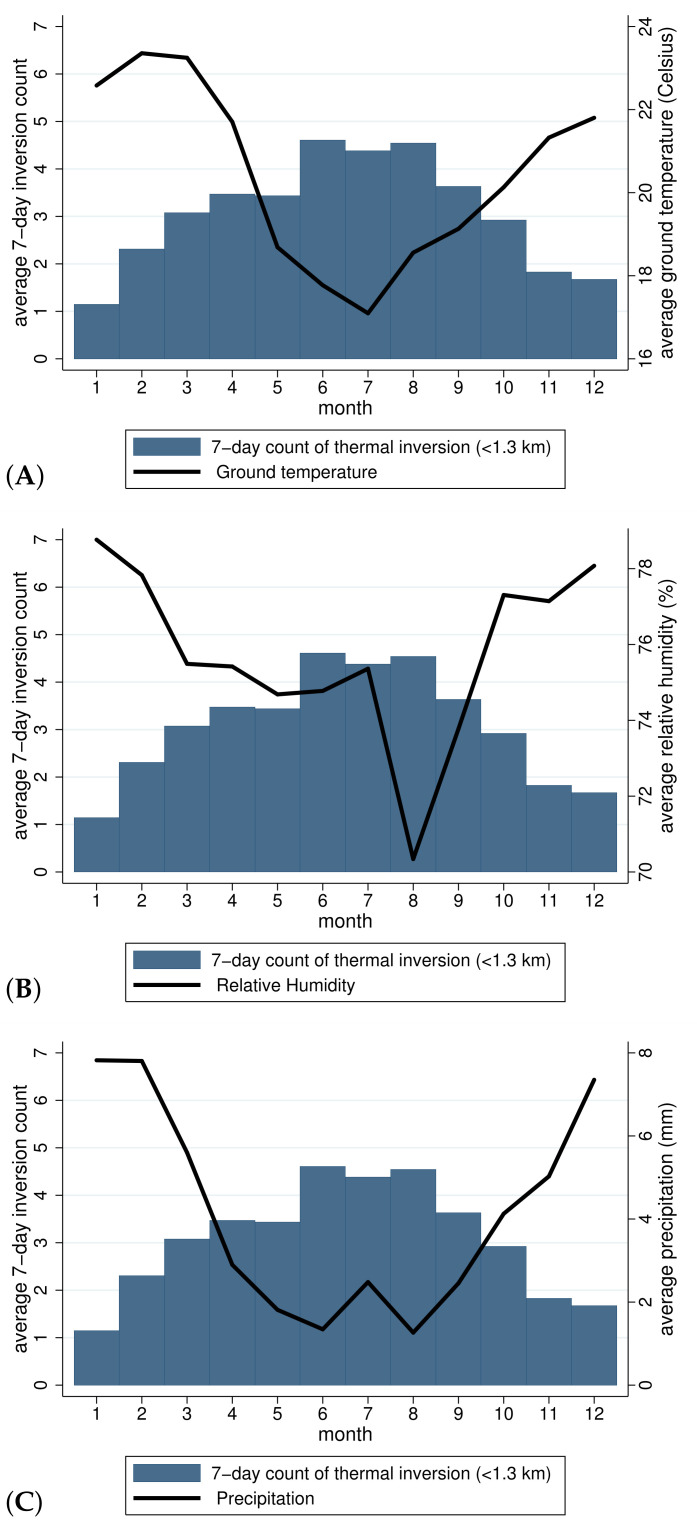
Average number of thermal inversions in a week (bars) and average weather (solid lines) in a week by calendar month. Inversions indicate those that occurred below 1.3 km. Weather variables are different in each panel, as follows: (**A**) ground temperature, (**B**) relative humidity, (**C**) rainfall.

To characterize the thermal inversion episodes in the SPMA, we plotted their relationships with weather conditions. Seasonality patterns associated with thermal inversions are clearly illustrated in Figure 1. For each calendar month, this figure shows the average number of inversions that occurs up to 1.3 km from the ground in a week (bars) and the weekly average of weather variables (solid line). The frequency of thermal inversion episodes and weather conditions are negatively correlated: during the spring (October to December) and the summer (January to March), average weekly temperature, humidity, and rainfall were higher than during the fall (April to June) and the winter (July to September), while the average number of weekly inversions was lower during warmer seasons than the colder ones.

### 4.5. Birth Data

Data came from individual records in the Brazilian Ministry of Health’s Usage Information System (DATASUS). Brazil vital records provide a complete coverage of births: in the mid-2000s, they had a coverage rate above 98% [47]. They provide information about newborns’ health, their mothers’ characteristics, and location of residence. We only included singletons in our analyses.

To link vital records to weather and pollution data, we proceeded in three steps. First, we defined the mothers’ locations of residence based on the information available in birth records. For newborns whose mothers resided in the São Paulo city, we recovered their districts of residence, and we used contour data from *Centro de Estudos da Metropole* (CEM/USP) to identify the district polygon centroid. Second, for infants in the capital with unidentified districts, we used the weighted polygon centroid employing 2000 census population as weights for each district listed on the CEM/USP data. Third, for newborns who resided at birth in municipalities outside São Paulo city, we used the population centroid. We identified 135 locations of residence (97 districts in the capital and 38 municipalities).

We proceeded to calculate the distance between each population-centroid and a monitor station using latitude and longitude location data. We employed locations that had weather stations within 20 miles in our analysis; this resulted in 123 locations of residence with measurable environmental conditions. Finally, we computed a weighted average of weather variables and pollutant concentrations weighting the data measured in each station by the inverse of the distance, from the station to the district/municipality centroid.

Outcomes of interest are birth weight and prematurity. Weight at birth was measured continuously in grams and as indicators for low or very low birth weight (<2500 grams or <1500 grams, respectively). Gestational age at birth was coded in categories in Brazilian birth certificates. We defined indicators for preterm (<37 gestational weeks) and very preterm (<32 gestational weeks) births. To reduce computational demands, we collapsed observations at the individual level by date of live birth and location of residence. Our final sample included 313,286 location–date cells (which corresponded to 90% of all possible date–location cells; 10% of the cells did not have a recorded live birth).

In Table A2, Panel A shows descriptive statistics of health outcomes. Newborn weight on average was 3.2 kilograms; 8 per 100 infants were low birth weight, and in 1 in 100, weight at birth was less than 1500 grams. Prematurity was found in almost 7 out of 100 newborns; 1 in 100 infants were very premature. Panel B focuses on covariates that characterize mother and child sociodemographic characteristics. Mothers in our sample were on average 25 years old at the time of delivery and gave birth to a white child. In 41% of the cases, the child was a first born. In terms of maternal education, 18% of mothers in our sample had more than high school, and the vast majority of them completed between 8 and 11 years of education (53%). Finally, Panel C presents the means and S.D. of the inversion counts used in the empirical strategy; they coincide with those in Table A1, but the represented averages are weighted by the incidence of live births in this case.

## 5. Empirical Specifications

We conducted two sets of estimations. Formally, we first tested the pollution effects of thermal inversion episodes, which are akin to a first-stage relationship. Our second set of estimations links thermal inversions to health outcomes and estimates an intention-to-treat effect. The existence of pollutants not included in our data that are linked to thermal inversions and the recurrence of missing pollution data, limits the interpretation of the effects of each particular pollutant using inversions as an instrument of it. Many pollutants were locked in an inversion episode; these pollutants may be correlated with each other, and they may also have a direct effect on infants’ health or economic activity. Thus, the unobservables in a second-stage regression for an instrumental variable estimation would be correlated to the outcome and to the instrument.

To estimate the connection between inversions and pollution, we used observations at the station (s)-date (t) level and estimated alternative versions of the following model:

(1) $$\begin{array}{c} Poll_{st}=\beta_{0}+\beta_{1}\;\;Inv_{t}+X_{st}^{\prime }\;\;\gamma +\mu_{ws}+\mu_{y}+\epsilon_{st} \end{array}$$

where $Poll_{st}$ is an average daily pollution concentration from date t−6 to *t* measured in station *s* location. We explore readings of five pollutants: PM_10_, CO, O_3_, NO_x_, and SO_2_. $Inv_{t}$ indicates the number of inversion episodes that occurred from date t−6 to *t*. In the auxiliary analysis, we ran non-parametric models, which suggested that the concentration of pollutants increased linearly with the number of inversions. To ease the interpretation of the effects across pollutants, we transformed the seven-day averages to be expressed in S.D. units based on a whole variation in the sample.

The vector $X_{st}$ includes cubic polynomials in weekly average temperature, weekly maximum temperature, weekly minimum temperature, weekly average humidity, direction of wind (fixed octants), and weekly average rainfall; an indicator for having at least one date between t−6 and *t* with missing inversion data; and the count of the number of dates in the past week without inversion data. This vector also includes measures of traffic sluggishness/intensity (measured as linear km of stop-and-go traffic, in ln scale) and of fuel-type utilization (measured as share of gasoline over all vehicular fuel consumed in the past three months). $\mu_{ws}$ are station-by-week of the year fixed effects that control for location-specific seasonality effects; $\mu_{y}$ are year fixed effects, that account for changes over time. We use two-way clustering of standard errors at the station and date level to account for potential spatial correlation and common weather trends. To explore dynamic effects inversions, we also estimated augmented models, which included lagged moving-week counts of inversions and lagged moving-week average weather conditions.

Our specification to estimate effects on health at birth is similar to Equation (1), but one that includes many lags to cover the full duration of a normal pregnancy. This constitutes a distributed lag model, using data aggregated at the location and date of birth level, and following [3] to include lags for 38 weeks from the recorded birth date. That is, for births in location of residence *l* on date *t*, we run,

(2) $$\begin{array}{c} H_{lt}=\sum_{s=0}^{38}\alpha_{s}Inv_{t-7s}+\sum_{s=0}^{38}X_{l,t-7s}^{\prime }\;\;\delta_{s}+\rho_{wl}+\rho_{y}+\eta_{lt} \end{array}$$

where $H_{lt}$ is an average birth outcome for births in location *l* on date *t*. $Inv_{t-7s}$ are the counts of the thermal inversion episodes for the week leading up to date t−7s, where *s* is measured in weeks and *t* is measured in days. Vectors of covariates for each lagged week are defined as in Equation (1); they also include location–birth date averages of maternal characteristics (education, age, and marital status), parity (first born and higher parity), and infant gender and race. Lastly, we control for location-by-week of the year fixed effects and year of birth fixed effects. Regressions are weighted by the number of births in each location–date cell.

From Equation (2), we recover many week-specific estimates that might be individually imprecise and unwieldy to report [48,49]. Thus, we report 13-week sums of the coefficients that correspond to the last, second-to-last, and third-to-last periods before birth—alike trimesters of gestation,

(3) $$\begin{array}{c} \alpha_{\left\{\underline{T},\bar{T}\right\}}\equiv \sum_{s=\underline{T}}^{\bar{T}}\alpha_{s} \end{array}$$

where the periods $\left\{\underline{T},\bar{T}\right\}$ are {0,12}, {13,25}, and {26,38}. Each of these coefficients indicates the effect of an increase of one unit in the number of thermal inversions in every week during periods of approximately three months. Defining exposure backwards from the date of birth is not ideal: the timing and length of pregnancy are endogenous. For some premature infants, the 38-weeks period might include weeks before their conception, and those who are exposed to inversions since early in their time *in utero* might be positively selected because they were not born prematurely. As such, estimates from what would correspond to the first three months of pregnancy should be interpreted with caution.

The key identifying assumption to get consistent estimates of $\alpha_{s}$ is that conditional on the weather controls and set of time and location fixed effects, no other unobserved factor influences inversions and health at birth. Our model controls for location-specific seasonality of birth and inversions, and it yields conservative estimates compared to controlling for common seasonality across locations.

## 6. Results

### 6.1. Effects of Thermal Inversions on Air Pollution

Table 1 presents findings from estimating Equation (1). Panel A shows that one additional inversion increases contemporaneous concentration of PM_10_ by 0.05 of one S.D. (or approximately 0.84 μg/m${}^{3}$). That corresponds to an increase of 2% of the average weekly concentration of PM_10_ in the sample. The exposure to CO, NO_x_, and SO_2_ also increases by similar amounts within the 0.02 to 0.04 of one S.D. range. For O_3_, we observe a positive, but statistically insignificant effect. These estimates indicate a large impact of inversions relative to the pollution generated by increased congestion. For example, the SO_2_ reading increases with an additional inversion by the same amount it would increase with 14% heavier traffic (given by the ratio of the two coefficients in Panel A).

In Panel B, we turn to a distributed lag formulation. These estimates indicate that thermal inversions have distinct, longer-term impacts on some pollutants. PM_10_, CO, and NO_x_ readings in the current seven-day period are shown to be impacted by inversions occurring up to two weeks before. Meanwhile SO_2_ and O_3_ seem to be affected only contemporaneously. Panel C wraps this analysis by estimating standard errors for the sum of these impacts over the 28-day window studied. It confirms stronger impacts of inversion over pollution exposure, with most cumulative effects from the lagged structure being more than twice as large as in the ones computed in the contemporaneous model (Panel A).

We extend the analysis in Table 2 to illustrate the close relation between the pollution generated by vehicular traffic and the trapping of pollutants near to the surface promoted by inversions. We do so in two ways. First, we examine pollution concentrations within weeks depending on inversions happening on weekend or workdays. The assumption is that during the workday, inversion traps pollution generated by heavier traffic than during the weekend (when traffic is lighter). Panel A confirms this logic showing that workday inversions are systematically more likely to increase pollution exposure (with the exception of O_3_). Alternatively, in Panel B, we examine the impact of inversions as a function of gasoline utilization. We see that inversions have larger impact on pollution exposure in period in which more pollution is generated by the increased reliance of vehicular usage of gasoline rather than ethanol.

Table A3 presents robustness checks using specifications with different sets of seasonality and time fixed effects. We find that estimates are mostly insensitive to the specification with the only exception being results for O_3_ readings, which become significant in a few cases. Overall, our main model seems to yield more conservative estimates than other specifications presented in Table A3. Compared to estimates of the accumulation of pollutants due to inversion formation in other papers looking at Sweden or Mexico [1,4], our estimates are on the lower tail of the distribution of effects as well.

To further explore the impact of inversions and pollution by traffic levels, in Table A4, we show results using our preferred specification and dividing the sample by rolling-weeks with more than one extreme-traffic day or without these days. An extreme-traffic day is defined as the top-quartile of the traffic distribution over the 2002–2009 period. Our findings document some differences in the impact of inversion over pollution between days with and without extreme traffic, particularly with certain pollutants for which motor-vehicle combustion engine residues are a bigger contributor. However, given the precision of the estimates, we cannot rule out these are indeed equal to one another.

Table A5 shows results stratified by periods. The differences across periods for the impact of inversions are not monotonic likely reflecting the fact that between 2005 and 2007, the volume of cars increases at a faster pace than the switch to ethanol (reinforced by economic boom), and once ethanol becomes a dominant fuel option (and the increase in the volume of vehicles stabilizes) the measured effects go down. In Table A6, we run our main specification using the wild bootstrap test, considering clusters at the station level and show that conclusions are not changed, and results are practically the same.

### 6.2. Effects of Thermal Inversions on Infant Health

Panel A in Table 3 presents estimates from Equation (2). We report full-sample estimates of the 13-week sums of coefficients for each of the birth outcomes. We find that an increase of one additional thermal inversion per week during the last 13 weeks of gestation leads to a reduction in birth weight of 23.3 grams (column 1). For 13–25 and 26–38 weeks before birth, an increase in the number of inversions have negative, but statistically insignificant and economically small effects, although their differential effects might be related to concerns about selection from prematurity.

The impact on birth weight is felt at the lower tail of the birth weight distribution: recurrent inversions in the last period of gestation increase the incidence of low birth weight by 7.3% (an effect of 0.60 per 100 on a base risk of 8.2 per 100) and raise the incidence of very low birth weight by 22.5% (an effect of 0.27 per 100 on a base level of 1.2 per 100). Columns 4 and 5 show that the effect of inversions on birth weight might be explained, at least in part, by their influence on the length of gestation. Rates of preterm birth and very preterm births increase by 1.0 and 0.36 per 100 on a risk base of 7.3 and 1.2 per 100, respectively. Interestingly, for prematurity rates, inversions happening earlier in the gestation are also found to have significant impacts. Overall, these negative effects highlight that the concentration of pollutant caused by the formation of inversions deteriorates newborns’ health. In Panel B of Table 3, we examine the robustness of these findings to the exclusion of covariates. Estimates show that our main results are robust to not controlling for location–birth date averages of maternal characteristics (education, age, and marital status), parity (first born and higher parity), and infants’ gender and race.

Does thermal inversion formation affect fetal survival? We answer this question by studying the effect on the number of live births. This approach provides a proxy for survival *in utero* and has been used in previous studies (for example, [3,50]). Using the number of births as a broader proxy for survival relies on the assumption that the formation of thermal inversions (conditional on weather conditions) is unrelated to the number of conceptions. It allows to overcome two drawbacks related to using reported stillbirths. First, only fetal death at 20 or more weeks of gestation are required to be reported as fetal death. Losses prior to 20 weeks are classified as miscarriages, and reliable data on miscarriages are rarely available. A change in fetal health would be underestimated because stillbirths may be underreported and, even if all occurrences are correctly observed, it would only account for losses later in a pregnancy. Second, a shock may move the distribution of fetal losses to cross the 20 weeks threshold [51]. Then, a negative health shock may be followed by a decrease in the number of reported fetal deaths because some of previously reported fetal deaths do not survive until the reporting threshold, even though this would not indicate an improvement in fetal health. A negative shock may also raise the occurrence of fetal deaths by influencing fetal health and *in utero* surviving. Disentangling the magnitude of these effects seems implausible making the sign of the bias unknown.

Column 1 of Table 4 presents estimates for the total number of live births. Because there are zero births for some location–date cells (in around 10% of them), we transform this outcome with the inverse hyperbolic sine. This allows us to interpret coefficients in exactly the same way as a standard logarithmic transformation (i.e., approximating percent changes). We find that a sustained increase of one thermal inversion per week occurring in any of the 13 weeks periods decreases significantly the number of live births. Specifically, for the last gestational period, there is a 24.8% reduction in the number of live births. Compared to the average number of births, this estimate indicates that one less infant is being born per municipality-date. The respective reduction from inversions in earlier periods of potential pregnancies are 14.6 and 12.4%. Overall, we find evidence that thermal inversions likely increase fetal death or difficult pregnancy viability. The estimate may also be capturing families’ avoidance responses by migrating outside the SPMA during pregnancy. While we cannot account for these responses on our administrative data, the effect is likely negligible considering that the composition of births does not change dramatically. We show that in the other columns of Table 4. Fetal mortality is likely impacting older mothers more strongly, which would explain the reduction in mothers’ average age when inversions increase (and early parity births as a consequence). Socioeconomic differences in the form of racial or maternal education are not observed, indicating that increased pollution affects all groups of mothers. We also see no difference in the function of the child’s gender (live births of boys and girls are equally less likely to occur).

Table A7 presents results stratified by periods. Similarly to the impact of inversions on pollution (Table A5), we observe that differences across periods are not monotonic for birth outcomes. The impact of thermal inversions on health at birth seem larger between 2005 and 2007 when the SPMA faced a faster increase in the volume of cars that the switch to ethanol.

To place the magnitude of our estimates in the literature, we translate our results into the effect of increasing one unit of a pollutant on birth weight. Because thermal inversion episodes affect the concentration of different pollutants and they correlate with each other, we refrain form referring to this quantity as the effect of each pollutant on health at birth. Adding lags in Equation (1), we estimate that, in a period of 13 weeks, an additional thermal inversion per week increases PM_10_ by up to 2.1 μg/m${}^{3}$ and CO by up to 0.04 ppm in that week. Combining this with our main results on Table 3, we calculate a 0.80 (44) grams reduction in birth weight per exposure to a week with one additional unit of PM_10_ (CO) during the final three months of pregnancy.

While the evidence on the effect of pollution on health at birth is growing, many of these studies focus on infant mortality, and only a few papers that look at health at birth offer a measure of the effect in terms of unit of pollutants. For instance, [40] study the influence of pollution in health at birth for mothers in New Jersey over the 1990s using a model that controls for place-by-season and family characteristics constant over time. They find a reduction of birth weight of 0.40 grams per unit of PM_10_ during the last trimester of gestation. Ref. [2] use mother fixed effects models and control for air quality alerts in Chile to estimate reductions of 16 grams per unit of CO ppm.

In all, our estimates are large compared to the literature. These differences may be explained by the fact that our estimates measure the effect of an additional thermal inversion on health at birth. Inversions trap many pollutants and likely result in a larger effect than in contexts without inversions, so attributing it all to PM10 is likely overestimating its impacts. Moreover, previous studies employed models that eliminate confounding from seasonality and composition, but they do not address confounding concerns from economic activity. Thus, the economic confounding may explain the differences in our estimates with previous studies. Lastly, given the heterogeneity of the effects we estimate (see Section 6.3), with Blacks being more strongly affected, there is room to conceive that in our context, access to care (based on socioeconomic status (SES)) may also magnify impacts.

### 6.3. Heterogeneity of impacts

Because health at birth differ by mothers’ and infants’ characteristics, and the accumulation of pollution may differ by the SES of different locations, we explore the effect of thermal inversion formation in the last 13 weeks of pregnancy across subgroups of population. Table 5 presents our results for male and female babies. Overall, males experienced worse effects on their health at birth compared to females, which is compatible with extensive medical literature on the fetal development trajectories and male sensitivity to external factors. In particular, we find that thermal inversions during the last period of gestation lead to a reduction on birth weight for males that almost double the effect on females (i.e., an additional inversion per week reduces birth weight by 35 grams in males and by 16 grams for girls). When using the child’s race (as a proxy for SES), we find that inversion formation has larger effects for black babies (Table 6, Panel B) than for white ones (Panel A). These findings suggest that SES may play an additional role in insulating fetal development from additional pollution exposure promoted by inversions.

## 7. Conclusions

Urban air pollution is one of the most critical issues worldwide. Growth in urban transportation and congestion are key elements behind that. A growing number of studies focused on developed countries have shown that prenatal exposure to air pollution harms health at birth and increases infant mortality. Central differences between developed and developing countries limit the understanding of consequences of air pollution in the latter based on results from richer countries. Thus, the magnitude and scope of the impact of exposure to air pollution on infants’ health in less-developed countries still remain unclear.

In this paper, we used data from one of the largest urban conglomerates, the metropolitan area of São Paulo. To avoid multiple confounders embedded on variations in pollution generation activities, we took advantage of the meteorological phenomenon of thermal inversion, which arguably exogenously locks pollutants closer to the ground. Therefore, we examined the increased exposure to commonly generated pollution over birth outcomes. We find that exposure to inversion episodes during the last three months of gestation decreases birth weight, increases the chances of prematurity, and greatly affects fetal survival. These results are strongly "robust" in regard to multiple specification checks, and they are statistically significant. Overall, the suggestion is that air pollution harms human capital in its earliest stage, *in utero*, and it may have lasting negative consequences on new generations, requiring local authorities to create environmental regulations and public health initiatives focused on improving prenatal care services.

## Figures and Tables

**Table 1:** Mean daily pollutant concentrations (in S.D. units) across São Paulo Metro and incidence of thermal inversions, 2002 to 2009.

	— Mean Concentrations from Day *t* to Day t−6 —				
	PM_10_	CO	NO_x_	SO_2_	O_3_
	(1)	(2)	(3)	(4)	(5)
Panel A: inversions under 1.3 km of altitude (relative to sea-level)—contemporaneous impacts					
Average traffic sluggishness from day *t* to day t−6 (*Ln* km)	0.176 ***	0.285 ***	0.208 ***	0.278 **	−0.169 ***
	(0.034)	(0.035)	(0.057)	(0.083)	(0.045)
# Inversion-days from day *t* to day t−6	0.050 ***	0.031 ***	0.023 ***	0.040 ***	0.014
	(0.007)	(0.006)	(0.006)	(0.010)	(0.009)
Panel B: inversions under 1.3 km of altitude (relative to sea-level)—distributed lags					
# Inversion-days from day *t* to day t−6	0.058 ***	0.028 ***	0.023 ***	0.043 ***	0.026 ***
	(0.006)	(0.006)	(0.007)	(0.012)	(0.007)
# Inversion-days from day t−7 to day t−13	0.049 ***	0.026 ***	0.021 ***	0.017	0.013
	(0.007)	(0.005)	(0.006)	(0.012)	(0.008)
# Inversion-days from day t−14 to day t−20	0.017 **	0.014 **	0.012 *	0.008	0.001
	(0.006)	(0.005)	(0.006)	(0.008)	(0.008)
# Inversion-days from day t−21 to day t−27	0.003	0.005	−0.005	0.001	0.011
	(0.007)	(0.006)	(0.006)	(0.009)	(0.007)
Panel C: inversions under 1.3 km of altitude (relative to sea-level)—cumulative/dynamic impacts (28-days)					
Cumulative impact of inversion in seven-day window	0.127 ***	0.073 ***	0.051 **	0.068 **	0.051 **
	(0.018)	(0.015)	(0.017)	(0.029)	(0.021)
Observations (Panel A)	52,147	31,568	19,021	12,604	31,396
Observations (Panels B and C)	51,758	31,337	18,871	12,464	31,162
Reference values (Pollutant S.D.)	16.8 μg/m${}^{3}$	0.57 ppm	57.0 ppb	5.7 μg/m${}^{3}$	12.5 μg/m${}^{3}$

Robust standard errors in parentheses are clustered in two ways: station and day. Coefficients on inversion indicate the effect of one additional inversion within the seven-day window of time. Traffic sluggishness is measured as a daily average in past seven-day window; weekends and holidays have values set to zero. Only inversions under 1.3 km of altitude (relative to sea-level) are accounted for. The average seven-day window has 3.1 inversions (with the sample having a S.D. of 1.8). All regressions include as controls temperature (average, maximum, and minimum), relative humidity (average, maximum, and minimum), and rainfall (total), all as thirdorder polynomials. Share of gasoline consumption over all vehicular fuel consumption in the past three months is included. Fixed-effects at year and week-of-year station levels are accounted for. Models use observations with a missing count of inversions (seven-day count of inversions is set to zero) and include a binary flag missing data as well as a counter of days with missing information. Distributed-lags models include lags for all controls. *** significant at 1% level, ** significant at 5% level, * significant at 10% level.

**Table 2:** Mean daily pollutant concentrations (in S.D. units) across São Paulo Metro and incidence of thermal inversions, contemporaneous impacts, 2002 to 2009.

	— Mean Concentrations from Day *t* to Day t−6 —				
	PM_10_	CO	NO_x_	SO_2_	O_3_
	(1)	(2)	(3)	(4)	(5)
Panel A: differential impact by weekday/weekend occurrence					
# Inversion-days from day *t* to day t−6	0.018	0.011	0.020 *	0.023	0.017
	(0.012)	(0.010)	(0.011)	(0.014)	(0.014)
# Workday inversion-days from day *t* to day t−6	0.044 ***	0.028 **	0.004	0.023 **	−0.003
	(0.013)	(0.010)	(0.010)	(0.010)	(0.014)
Panel B: differential impact by fuel type utilization					
# Inversion-days from day *t* to day t−6	0.051 ***	0.033 ***	0.022 ***	0.037 ***	0.015 *
	(0.007)	(0.007)	(0.006)	(0.008)	(0.008)
Monthly share of gasoline on total vehicular fuel consumption × ...					
# Inversion-days from day *t* to day t−6	0.157 ***	0.103 *	−0.012	0.211 ***	0.054
	(0.052)	(0.049)	(0.039)	(0.054)	(0.069)
Panel C: differential impact by fuel type utilization (holding constant interaction with traffic sluggishness)					
# Inversion-days from day *t* to day t−6	0.053 ***	0.034 ***	0.022 ***	0.038 ***	0.019 **
	(0.007)	(0.007)	(0.006)	(0.009)	(0.009)
Monthly share of gasoline on total vehicular fuel consumption × ...					
# Inversion-days from day *t* to day t−6	0.124 **	0.096 *	−0.009	0.233 ***	−0.047
	(0.053)	(0.052)	(0.045)	(0.046)	(0.066)
Observations	52,147	31,568	19,021	12,604	31,396

Robust standard errors in parentheses clustered in two ways: station and day. Coefficients on inversion indicate the effect of one additional inversion within the seven-day window of time. Traffic sluggishness is measured as a daily average in past seven-day window; weekends and holidays have values set to zero. Only inversions under 1.3 km of altitude (relative to sea-level) are accounted for. The average seven-day window has 3.1 inversions (with the sample having a S.D. of 1.8). All regressions include as controls temperature (average, maximum, and minimum), relative humidity (average, maximum, and minimum), and rainfall (total), all as third-order polynomials. Share of gasoline consumption over all vehicular fuel consumption in the past three months is included. Fixed-effects at year and week-of-year station levels are accounted for. Models use observations with a missing count of inversions (seven-day count of inversions is set to zero) and include a binary flag missing data as well as a counter of days with missing information. Interaction with fuel composition and total fuel consumption are included centered at the sample mean. *** significant at 1% level, ** significant at 5% level, * significant at 10% level.

**Table 3:** Birth outcomes and inversion exposure.

	Birth Weight	Low Birth Weight	Very Low Birth Weight	Premature	Very Premature
	(Grams)	(per 100)	(per 100)	(per 100)	(per 100)
	(1)	(2)	(3)	(4)	(5)
Panel A: base model—controls for child and mother covariates					
# Inversion-days/week in week 0–12 before birth	−23.345 ***	0.596 **	0.271 **	1.004 ***	0.357 ***
	(6.980)	(0.283)	(0.136)	(0.317)	(0.111)
# Inversion-days/week in week 13-25 before birth	−1.500	−0.426	0.104	0.858 ***	0.182 **
	(5.391)	(0.309)	(0.114)	(0.295)	(0.084)
# Inversion-days/week in week 26-38 before birth	1.086	−0.314	0.058	−0.050	−0.029
	(5.382)	(0.319)	(0.107)	(0.315)	(0.105)
Panel B: model without child and mother covariates					
# Inversion-days/week in week 0–12 before birth	−25.541 ***	0.608 **	0.270 *	1.005 ***	0.362 ***
	(7.345)	(0.282)	(0.136)	(0.314)	(0.111)
# Inversion-days/week in week 13-25 before birth	−2.047	−0.415	0.113	0.851 ***	0.181 **
	(5.704)	(0.318)	(0.112)	(0.302)	(0.086)
# Inversion-days/week in week 26-38 before birth	−0.828	−0.271	0.072	−0.058	−0.027
	(5.395)	(0.317)	(0.109)	(0.307)	(0.106)
Observations	313,286	313,286	313,286	313,286	313,286

Robust standard errors in parentheses clustered in two ways: location and day. Observations are at location–date level and are weighted by the size of the local birth cohort in that location–day. Coefficients indicate the effect of one additional inversion in each seven-day count of inversions during the interval of weeks indicated. Only inversions under 1.3 km of altitude (relative to sea-level) are accounted for. The average seven-day window has 3.1 inversions (with the sample having a S.D. of 1.8). All regressions include as controls temperature (average, maximum, and minimum), relative humidity (average, maximum, and minimum), and rainfall (total), all as third-order polynomials. Share of gasoline consumption over all vehicular fuel consumption in the past three months is included, as well as a measure of traffic sluggishness/intensity. Traffic sluggishness is measured as a daily average in past seven-day windows; weekends and holidays have values set to zero. Fixed-effects at year of birth and week-of-year location levels are accounted for. Models use observations with a missing count of inversions (seven-day count of inversions is set to zero) and include a binary flag missing data as well as a counter of days with missing information. Regressions in Panel A include control for location–birth date averages of maternal characteristics (education, age, and marital status), parity (first born and higher parity), and infants’gender and race. *** significant at 1% level, ** significant at 5% level, * significant at 10% level.

**Table 4:** Birth cohorts size and composition in relation to inversion exposure.

	Nr births	——————————— Average Composition ———————————						
	(Inv. Hip. Sine)	Mom Age	White	Black-Brown	Male	Mom College+	Mom Married	First Born
	(1)	(2)	(3)	(4)	(5)	(6)	(7)	(8)
# Inversion-days/week in week 0–12 before birth	−0.248 ***	−0.380 **	−0.017	0.010	−0.002	−0.001	−0.025	0.016 *
	(0.056)	(0.150)	(0.017)	(0.015)	(0.006)	(0.012)	(0.016)	(0.010)
# Inversion-days/week in week 13–25 before birth	−0.146 **	0.073	−0.018	0.014	−0.004	0.015	−0.018	−0.011
	(0.069)	(0.147)	(0.017)	(0.011)	(0.006)	(0.016)	(0.015)	(0.010)
# Inversion-days/week in week 26–38 before birth	−0.124 *	−0.425 ***	−0.023	0.008	0.000	−0.005	−0.007	−0.010
	(0.064)	(0.150)	(0.015)	(0.011)	(0.004)	(0.015)	(0.012)	(0.010)
Observations	341,145	313,286	313,286	313,286	313,286	313,286	313,286	313,286

Robust standard errors in parentheses clustered in two ways: location and day. Observations are at location–date level and are weighted by the size of the local birth cohort in that location–day. Coefficients indicate the effect of one additional inversion in each seven-day count of inversions during the interval of weeks indicated. Only inversions under 1.3 km of altitude (relative to sea-level) are accounted for. The average seven-day window has 3.1 inversions (with the sample having a S.D. of 1.8). All regressions include as controls temperature (average, maximum, and minimum), relative humidity (average, maximum, and minimum), and rainfall (total), all as third-order polynomials. Share of gasoline consumption over all vehicular fuel consumption in the past three months is included as well as a measure of traffic sluggishness/intensity. Traffic sluggishness is measured as a daily average in past seven-day windows; weekends and holidays have values set to zero. Fixed-effects at year of birth and week-of-year location levels are accounted for. Models use observations with a missing count of inversions (seven-day count of inversions is set to zero) and include a binary flag missing data as well as a counter of days with missing information. *** significant at 1% level, ** significant at 5% level, * significant at 10% level.

**Table 5:** Outcomes from the last 13 weeks of exposure by the child’s sex.

	Birth Weight	Low Birth Weight	Very Low Birth Weight	Premature	Very Premature
	(Grams)	(per 100)	(per 100)	(per 100)	(per 100)
	(1)	(2)	(3)	(4)	(5)
Panel A: boys only					
# Inversion-days/week in week 0–12 before birth	−34.951 ***	0.443	0.307 *	1.025 ***	0.319 *
	(9.507)	(0.423)	(0.184)	(0.371)	(0.182)
Observations	275,589	275,589	275,589	275,589	275,589
Panel B: girls only					
# Inversion-days/week in week 0–12 before birth	−16.161 *	0.780 *	0.247	1.041 *	0.417 **
	(9.541)	(0.460)	(0.194)	(0.531)	(0.179)
Observations	271,866	271,866	271,866	271,866	271,866

Robust standard errors in parentheses clustered in two ways: location and day. Observations are at location–date level and are weighted by the size of the local birth cohort in that location–day. Coefficients indicate the effect of one additional inversion in each seven-day count of inversions during the interval of weeks indicated. Only inversions under 1.3 km of altitude (relative to sea-level) are accounted for. The average seven-day window has 3.1 inversions (with the sample having a S.D. of 1.8). All regressions include as controls temperature (average, maximum, and minimum), relative humidity (average, maximum, and minimum), and rainfall (total), all as third-order polynomials. Share of gasoline consumption over all vehicular fuel consumption in the past 3-months is included as well as a measure of traffic sluggishness/intensity. Traffic sluggishness is measured as a daily average in past seven-day windows; weekends and holidays have values set to zero. Fixed-effects at year of birth and week-of-year location levels are accounted for. Models use observations with a missing count of inversions (seven-day count of inversions is set to zero) and include a binary flag missing data as well as a counter of days with missing information. Regressions include control for location–birth date averages of maternal characteristics (education, age, and marital status), parity (first born and higher parity), and infants’ race. *** significant at 1% level, ** significant at 5% level, * significant at 10% level.

**Table 6:** Outcomes from last 13 weeks exposure by child’s race.

	Birth Weight	Low Birth Weight	Very Low Birth Weight	Premature	Very Premature
	(Grams)	(per 100)	(per 100)	(per 100)	(per 100)
	(1)	(2)	(3)	(4)	(5)
Panel A: white children only					
# Inversion-days/week in week 0–12 before birth	−18.176 *	0.148	0.225	1.007 **	0.366 **
	(10.143)	(0.470)	(0.210)	(0.504)	(0.149)
Observations	281,026	281,026	281,026	281,026	281,026
Panel B: black children only					
# Inversion-days/week in week 0–12 before birth	−32.730 **	0.620	0.409 *	1.057	0.863 ***
	(13.794)	(0.720)	(0.238)	(1.028)	(0.324)
Observations	181,971	181,971	181,971	181,971	181,971

Robust standard errors in parentheses clustered in two ways: location and day. Observations are at location–date level and are weighted by the size of the local birth cohort in that location–day. Coefficients indicate the effect of one additional inversion in each seven-day count of inversions during the interval of weeks indicated. Only inversions under 1.3 km of altitude (relative to sea-level) are accounted for. The average seven-day window has 3.1 inversions (with the sample having a S.D. of 1.8). All regressions include as controls temperature (average, maximum, and minimum), relative humidity (average, maximum, and minimum), and rainfall (total), all as third-order polynomials. Share of gasoline consumption over all vehicular fuel consumption in the past three months is included as well as a measure of traffic sluggishness/intensity. Traffic sluggishness is measured as a daily average in past seven-day windows; weekends and holidays have values set to zero. Fixed-effects at year of birth and week-of-year location levels are accounted for. Models use observations with a missing count of inversions (seven-day count of inversions is set to zero) and include a binary flag missing data as well as a counter of days with missing information. Regressions include control for location–birth date averages of maternal characteristics (education, age, and marital status), parity (first born and higher parity), and infants’ gender. *** significant at 1% level, ** significant at 5% level, * significant at 10% level.

## Data Availability

Publicly available datasets were analyzed in this study. Data can be found here: https://datasus.saude.gov.br/, https://cetesb.sp.gov.br/ (accessed on 11 November 2021).

## Appendix A

**Table A1:** Descriptive statistics by station/rolling-week, 2002 to 2009.

	Mean	SD	Obs.
	(1)	(2)	(3)
Panel A: Pollutants (average within seven-day period)			
PM ${}_{10}$ μg/m${}^{3}$	41.18	16.76	52,147
CO ppm	1.23	0.57	31,568
NO${}_{x}$ ppb	77.47	56.99	19,022
SO${}_{2}$ μg/m${}^{3}$	10.89	5.72	12,608
O${}_{3}$ μg/m${}^{3}$	33.64	12.53	31,396
Panel B: Weather (average within seven-day period)			
Average temperature (Celsius)	20.45	2.66	102,270
Max temperature (Celsius)	25.85	3.16	102,270
Min temperature (Celsius)	16.62	2.68	102,270
Average relative humidity (%)	76.03	7.88	102,270
Max relative humidity (%)	92.25	5.64	102,270
Min relative humidity (%)	52.32	11.04	102,270
Average precipitation (milimeters)	4.10	4.65	102,270
Missing wind direction (share)	0.06	0.10	102,270
NNE wind (share)	0.10	0.07	102,270
ENE wind (share)	0.13	0.07	102,270
ESE wind (share)	0.15	0.10	102,270
SSE wind (share)	0.29	0.14	102,270
SSW wind (share)	0.07	0.05	102,270
WSW wind (share)	0.02	0.02	102,270
WNW wind (share)	0.08	0.07	102,270
NNW wind (share)	0.10	0.08	102,270
Panel C: Inversions (counts within seven-day period)			
Inversions	2.80	1.95	2,922
Weekday inversions	2.03	1.55	2,922
Panel D: Metro-area-level conditions (average within seven-day period)			
Traffic sluggishness (km)	61.88	15.26	2922
Share of gasoline on fuel consumption	0.66	0.14	2922

Observations (Obs.) correspond to the period 2002-2009 for the SPMA. Pollutants are measured by station-date, in an unbalanced panel (different pollutants have different panel structures, leading to different observations). Weather variables are based on interpolations for missing values, producing a balanced panel of stations and days. Inversions are based on one observation per day covering the entire metropolitan area. The same applies to traffic observations and fuel consumption.

**Table A2:** Descriptive statistics by live-birth/date, 2002 to 2009.

	Mean	SD	Obs.
	(1)	(2)	(3)
Panel A: Birth outcomes			
Birth weight (grams)	3153.2	202.49	313,286
Low birth weight (per 100)	8.20	10.43	313,286
Very low birth weight (per 100)	1.22	4.18	313,286
Premature (per 100)	7.26	10.10	313,286
Very premature (per 100)	1.16	4.10	313,286
Panel B: Demographic characteristics			
Maternal age at birth	26.49	2.66	313,286
Child white	0.56	0.24	313,286
Child black or brown	0.24	0.20	313,286
Child male	0.51	0.19	313,286
Maternal education more than high-school	0.18	0.19	313,286
Maternal education complete primary, high-school dropouts and graduates	0.53	0.22	313,286
Maternal education incomplete primary	0.23	0.17	313,286
Maternal education incomplete elementary	0.04	0.08	313,286
Married at time of birth	0.43	0.22	313,286
First born child	0.41	0.21	313,286
Panel C: Inversion exposure			
Inversions in past 7 days	2.91	1.92	313,286

Observations (Obs.) are number of location–date cells, and correspond to 2,140,984 unique births. All statistics are weighted by the size of the location–date live-birth cohort.

**Table A3:** Mean daily pollutant concentrations (in S.D. units) across São Paulo Metro and incidence of thermal inversions, 2002 to 2009—alternative specifications.

	— Mean Concentrations from Day *t* to Day t−6 —				
	PM_10_	CO	NO_x_	SO_2_	O_3_
	(1)	(2)	(3)	(4)	(5)
Panel A: Year and week-of-year × location FE’s (main model in text)					
Average traffic sluggishness from day *t* to day t−6 (*Ln* km)	0.176 ***	0.285 ***	0.208 ***	0.278 **	−0.169 ***
	(0.034)	(0.035)	(0.057)	(0.083)	(0.045)
# Inversion-days from day *t* to day t−6	0.050 ***	0.031 ***	0.023 ***	0.040 ***	0.014
	(0.007)	(0.006)	(0.006)	(0.010)	(0.009)
Panel B: Year, week-of-year, and location FE’s					
Average traffic sluggishness from day *t* to day t−6 (*Ln* km)	0.179 ***	0.285 ***	0.225 ***	0.270 **	−0.171 ***
	(0.034)	(0.036)	(0.058)	(0.078)	(0.045)
# Inversion-days from day *t* to day t−6	0.050 ***	0.032 ***	0.025 ***	0.038 ***	0.015 *
	(0.007)	(0.006)	(0.006)	(0.010)	(0.008)
Panel C: Week-of-year and year × location FE’s					
Average traffic sluggishness from day *t* to day t−6 (*Ln* km)	0.191 ***	0.302 ***	0.235 ***	0.271 ***	−0.184 ***
	(0.034)	(0.036)	(0.056)	(0.069)	(0.044)
# Inversion-days from day *t* to day t−6	0.044 ***	0.027 ***	0.021 ***	0.037 **	0.012
	(0.006)	(0.005)	(0.006)	(0.012)	(0.007)
Panel D: Bi-month × location FE’s and location-specific linear month-level trends					
Average traffic sluggishness from day *t* to day t−6 (*Ln* km)	0.133 ***	0.241 ***	0.222 ***	0.267 ***	−0.279 ***
	(0.031)	(0.034)	(0.056)	(0.056)	(0.042)
# Inversion-days from day *t* to day t−6	0.069 ***	0.053 ***	0.032 ***	0.048 ***	0.000
	(0.007)	(0.006)	(0.007)	(0.011)	(0.007)
Panel E: Week-of-year, and Month-year × location FE’s					
Average traffic sluggishness from day *t* to day t−6 (*Ln* km)	0.227 ***	0.326 ***	0.272 ***	0.267 ***	−0.277 ***
	(0.037)	(0.043)	(0.067)	(0.067)	(0.036)
# Inversion-days from day *t* to day t−6	0.041 ***	0.030 ***	0.017 **	0.044 ***	0.028 ***
	(0.006)	(0.006)	(0.006)	(0.008)	(0.006)
Panel F: Month-year, and Week-of-year × location FE’s					
Average traffic sluggishness from day *t* to day t−6 (*Ln* km)	0.200 ***	0.314 ***	0.256 ***	0.265 ***	−0.277 ***
	(0.037)	(0.039)	(0.066)	(0.075)	(0.036)
# Inversion-days from day *t* to day t−6	0.049 ***	0.036 ***	0.024 ***	0.043 ***	0.028 ***
	(0.008)	(0.007)	(0.006)	(0.007)	(0.008)
Observations	52,147	31,568	19,021	12,604	31,396

Panel A replicates results on Table 1. Regressions in other panels include fixed-effects at different levels as indicated on the table. *** significant at 1% level, ** significant at 5% level, * significant at 10% level.

**Table A4:** Mean daily pollutant concentrations (in S.D. units) across São Paulo Metro and incidence of thermal inversions, 2002 to 2009—strata by traffic intensity.

	— Mean Concentrations from Day *t* to Day t−6 —				
	${\mathrm{PM}}_{10}$	CO	${\mathrm{NO}}_{x}$	${\mathrm{SO}}_{2}$	$O_{3}$
	**(1)**	**(2)**	**(3)**	**(4)**	**(5)**
Panel A: Rolling-weeks with more than one extreme-traffic day					
# Inversion-days from day *t* to day t−6	0.048 ***	0.032 ***	0.031 ***	0.040 ***	0.014
	(0.008)	(0.008)	(0.007)	(0.009)	(0.011)
Observations	20,690	13,893	8285	4606	13,181
Panel B: Rolling-weeks without extreme-traffic days					
# Inversion-days from day *t* to day t−6	0.037 ***	0.024 *	0.005	0.033 **	0.027 **
	(0.012)	(0.014)	(0.011)	(0.013)	(0.011)
Observations	19,366	10,304	6394	5117	10,941

Robust standard errors in parentheses clustered in two ways: station and day. Coefficients on inversion indicate the effect of one additional inversion within the seven-day window of time. Only inversions under 1.3 km of altitude (relative to sea-level) are accounted for. The average seven-day window has 3.1 inversions (with the sample having a S.D. of 1.8). All regressions include as controls temperature (average, maximum, and minimum), relative humidity (average, maximum, and minimum), and rainfall (total), all as third-order polynomials. Share of gasoline consumption over all vehicular fuel consumption in the past 3-months is included. Fixed-effects at year and week-of-year station levels are accounted for. Models use observations with a missing count of inversions (seven-day count of inversions is set to zero) and include a binary flag missing data as well as a counter of days with missing information. Traffic sluggishness is measured as a daily average in past seven-day window; weekends and holidays have values set to zero. Regressions in Panel A include rolling-weeks with more than in extreme-traffic day (defined as top-quartile of traffic distribution over the 2002–2009 period); Panel B show regressions for the remaining rolling weeks. *** significant at 1% level, ** significant at 5% level, * significant at 10% level.

**Table A5:** Mean Daily Pollutant Concentrations (in S.D. Units) across São Paulo Metro and Incidence of Thermal Inversions, 2002 to 2009—strata by periods.

	— Mean Concentrations from Day *t* to Day t−6 —				
	PM_10_	CO	NO_x_	SO_2_	O_3_
	(1)	(2)	(3)	(4)	(5)
Panel A: 2002∓2004					
# Inversion-days from day *t* to day t−6	0.053 ***	0.027 **	0.007	0.043 **	0.005
	(0.012)	(0.011)	(0.010)	(0.013)	(0.012)
Observations	20,854	10,545	6634	5648	11,699
Panel B: 2005∓2007					
# Inversion-days from day *t* to day t−6	0.070 ***	0.043 ***	0.028 **	0.029	0.044 ***
	(0.011)	(0.010)	(0.010)	(0.021)	(0.014)
Observations	18,537	11,865	6543	4256	10,851
Panel C: 2008∓2009					
# Inversion-days from day *t* to day t−6	0.025 **	0.026 **	0.022 ***	0.033 **	−0.007
	(0.011)	(0.009)	(0.007)	(0.011)	(0.018)
Observations	12,752	9153	5844	2696	8842

Robust standard errors in parentheses clustered in two ways: station and day. Coefficients on inversion indicate the effect of one additional inversion within the seven-day window of time. Traffic sluggishness is measured as a daily average in past seven-day window; weekends and holidays have values set to zero. Only inversions under 1.3 km of altitude (relative to sea-level) are accounted for. The average seven-day window has 3.1 inversions (with the sample having a S.D. of 1.8). All regressions include as controls temperature (average, maximum, and minimum), relative humidity (average, maximum, and minimum), and rainfall (total), all as third-order polynomials. Share of gasoline consumption over all vehicular fuel consumption in the past 3-months is included. Fixed-effects at year and week-of-year station levels are accounted for. Models use observations with a missing count of inversions (seven-day count of inversions is set to zero) and include a binary flag missing data as well as a counter of days with missing information. In Panel A includes observations for the period 2002–2004, Panel B for 2005–2007, and Panel C for 2008–2009. *** significant at 1% level, ** significant at 5% level.

**Table A6:** Mean Daily Pollutant Concentrations (in S.D. Units) across São Paulo Metro and Incidence of Thermal Inversions, 2002 to 2009—Wild-Boostrap *p*-values.

	— Mean Concentrations from Day *t* to Day t−6 —				
	PM_10_	CO	NO_x_	SO_2_	O_3_
	(1)	(2)	(3)	(4)	(5)
Average traffic sluggishness from day *t* to day t−6 (*Ln* km)	0.179	0.285	0.225	0.271	−0.171
	[<0.001]	[<0.001]	[<0.001]	[<0.001]	[<0.001]
# Inversion-days from day *t* to day t−6	0.050	0.032	0.025	0.038	0.015
	[<0.001]	[<0.001]	[<0.001]	[0.024]	[0.072]
Observations	52,147	31,568	19,021	12,604	31,396

Wild-Bootstrap p-values (based in 249 replications) in brackets under estimates are based on station-level clusters. Coefficients on inversion indicate the effect of one additional inversion within the seven-day window of time. Traffic sluggishness is measured as a daily average in past seven-day window; weekends and holidays have values set to zero. Only inversions under 1.3 km of altitude (relative to sea-level) are accounted for. The average seven-day window has 3.1 inversions (with the sample having a S.D. of 1.8). All regressions include as controls temperature (average, maximum, andminimum), relative humidity (average, maximum, andminimum), and rainfall (total), all as third-order polynomials. Share of gasoline consumption over all vehicular fuel consumption in the past 3-months is included. Fixed-effects at year and week-of-year station levels are accounted for. Models use observations with a missing count of inversions (seven-day count of inversions is set to zero) and include a binary flag missing data as well as a counter of days with missing information. Distributed-lags models include lags for all controls.

**Table A7:** Inversions and Birth Outcomes—strata by periods.

	— Mean Concentrations from Day *t* to Day t−6 —				
	PM_10_	CO	NO_x_	SO_2_	O_3_
	(1)	(2)	(3)	(4)	(5)
Panel A: 2002–2004					
# Inversion-days/week in week 0–12 before birth	47.010	0.478	−0.185	2.209	−0.228
	(36.766)	(1.430)	(0.364)	(1.576)	(0.476)
# Inversion-days/week in week 13-25 before birth	−78.725 **	2.547 *	0.292	2.450	0.012
	(28.211)	(1.246)	(0.453)	(1.628)	(0.477)
# Inversion-days/week in week 26-38 before birth	−80.051 ***	2.937 **	0.314	3.942 **	−0.416
	(21.506)	(1.287)	(0.491)	(1.437)	(0.524)
Observations	90,596	90,596	90,596	90,596	90,596
Panel B: 2005–2007					
# Inversion-days/week in week 0–12 before birth	−63.550 ***	0.712	0.456	1.869	0.903 **
	(16.470)	(0.868)	(0.373)	(1.115)	(0.374)
# Inversion-days/week in week 13–25 before birth	−44.677 ***	−0.115	0.403 *	2.064 **	0.440
	(15.125)	(0.750)	(0.236)	(0.762)	(0.293)
# Inversion-days/week in week 26–38 before birth	−6.997	−0.799	−0.279	1.612 **	−0.000
	(14.049)	(0.728)	(0.257)	(0.704)	(0.237)
Observations	133,462	133,462	133,462	133,462	133,462
Panel C: 2008-2009					
# Inversion-days/week in week 0–12 before birth	−33.337	3.495	0.874	5.811 **	1.416 *
	(45.532)	(2.441)	(0.732)	(2.444)	(0.745)
# Inversion-days/week in week 13–25 before birth	−25.735	−0.841	−0.080	2.499	0.714
	(51.192)	(2.290)	(0.971)	(3.380)	(1.072)
# Inversion-days/week in week 26–38 before birth	74.706	−2.638	−0.953	−2.340	−0.233
	(46.828)	(2.803)	(1.016)	(2.784)	(0.911)
Observations	89,228	89,228	89,228	89,228	89,228

Robust standard errors in parentheses clustered in two ways: location and day. Observations are at location–date level and are weighted by the size of the local birth cohort in that location–day. Coefficients indicate the effect of one additional inversion in each seven-day count of inversions during the interval of weeks indicated. Only inversions under 1.3 km of altitude (relative to sea-level) are accounted for. The average seven-day window has 3.1 inversions (with the sample having a S.D. of 1.8). All regressions include as controls temperature (average, maximum, and minimum), relative humidity (average, maximum, and minimum), and rainfall (total), all as third-order polynomials. Share of gasoline consumption over all vehicular fuel consumption in the past 3-months is included as well as a measure of traffic sluggishness/intensity. Traffic sluggishness is measured as a daily average in past seven-day windows; weekends and holidays have values set to zero. Fixed-effects at year of birth and week-of-year location levels are accounted for. Models use observations with a missing count of inversions (seven-day count of inversions is set to zero) and include a binary flag missing data as well as a counter of days with missing information. *** significant at 1% level, ** significant at 5% level, * significant at 10% level.
